# The assessment of laboratory parameters in children with fever and febrile seizures

**DOI:** 10.1002/brb3.720

**Published:** 2017-05-15

**Authors:** Krystyna Gontko – Romanowska, Zbigniew Żaba, Paweł Panieński, Barbara Steinborn, Michał Szemień, Magdalena Łukasik – Głębocka, Krystian Ratajczak, Jacek Górny

**Affiliations:** ^1^ Department of Emergency Medicine Poznan University of Medical Sciences Poznan Poland; ^2^ Specialized Health Care Centre for Mother and Child Poznan Poland; ^3^ Department of Teaching Anesthesiology and Intensive Therapy Poznan University of Medical Sciences Poznan Poland; ^4^ Department of Developmental Neurology Poznan University of Medical Sciences Poznan Poland

**Keywords:** children, febrile seizures, fever, laboratory results

## Abstract

**Objective:**

The aim of the research paper was to assess selected laboratory results in children with fever without seizures and febrile seizure.

**Materials and Methods:**

The paper presents an analysis of a group of 306 children aged 6 months – 5 years who were admitted with diagnosed fever without seizures and febrile seizures in Specialized Health Care Centre for Mother and Child in Poznan between 1st January 2008 and 31st December 2009. Out of the diagnostics procedures performed in children the following ones were taken into consideration: BCC and CRP.

**Results:**

Of the analyzed group of 306 children, 59.48% were boys and 40.52% were girls. In the studied group 61.93% were boys and control group 56.15% were boys. Mean age of admitted children was 22 months. In the study group mean body temperature was 39.0°C and in the control group 38.6°C. A statistically significant difference was found between body temperature of study and control group (*p = *.005). The mean C‐reactive protein level in the study group was 15.73 mg/L and in the control group 58.20 mg/L. There was a statistically significant difference (*p < *.001). There was a statistically significant difference between the number of lymphocytes and neutrophils (*p < *.001). There was also a statistically significant difference between the number of hemoglobin, hematocrit and platelets.

**Conclusions:**

The study showed that children with FS, had statistically significant higher neutrophils level compared to those with fever without seizures. The number of lymphocytes was lower in children with FS than in children with fever without seizures.

## 1 INTRODUCTION

Through ages fever was subject of many publications. Fever is defined as a temperature above normal range as result of pyrogen concentration. In the XIX century Carl Liebermeister recognized the role of infectious factor, described clinical presentation and complications of fever. Fever in children can be complicated by febrile seizures (FS), which is observed in 2%–5% of children in the European population. Febrile seizures mostly occur in children between 6 months and 5 years of age. Body temperature is typically above 38°C. It is not due to acute disease of the nervous system. There is the division of FS into three groups, as follows: simple febrile seizures (SFS), simple febrile seizures plus (SFS+) and complex febrile seizures (CFS). Duration of SFS in less than 15 min, seizures are described as generalized tonic‐clonic. Simple febrile seizures plus are characterized by more than one attack of SFS, where no neurological abnormalities are found. On the other side, we have got CFS with prolonged duration (over 15 min), focal symptoms or multiple seizures occur in close succession.

Management of FS is administration of diazepam if seizures do not stop by themselves. Administration of antipyretic drugs (paracetamol, ibuprofen) decreases secondary symptoms to elevated body temperature and fever‐related discomfort. These drugs do not prevent FS (Gontko – Romanowska et al., 2016; Grygalewicz, 2009; Kozak, 2009; Wendorff, Wiśniewska, Piotrowicz, & Chamielec, 2009).

The aim of the research paper was to assess selected laboratory results in children with fever without seizures and FS in the Polish population.

## 2 MATERIALS AND METHODS

The paper presents an analysis of a group of 306 children aged 6 months – 5 years who were admitted because of fever without seizures and FS in Specialized Health Care Centre for Mother and Child in Poznan between 1st January 2008 and 31st December 2009.

Patient were divide into two groups: a study group consisting of 176 children (109 boys and 67 girls) with FS and a control group of 130 children (73 boys and 57 girls) with fever of unknown etiology without seizures.

In this retrospective research during the analysis of medical records the following laboratory results were taken into consideration:

- Blood cell count (BCC)—to conduct the assessment the following tests were performed: the number of leukocytes—WBC (G/L unit), neutrophils (%), monocytes (%), lymphocytes (%), hematocrit (%), hemoglobin (g/dl) and platelets count (thousands/ml),
- C‐reactive protein (CRP) concentration in mg/L.

These results were performed in children on admission with life threatening condition and acute phase of infectious disease.

STATGRAPHICS CENTURION XVI was used for statistical analysis. T‐test was run to compare two samples of data (study and control group). Pearson product moment test was used to analyze correlation between pairs of variables. *p* < .05 value was considered as statistically significant.

## 3 RESULTS

Of the analyzed group of 306 children, 59.48% (*n* = 182) were boys and 40.52% (*n* = 124) were girls. In the studied group 61.93% were boys and control group 56.15% were boys. Difference between groups wasn't statistically relevant (*p = *1.000).

Mean age of hospitalized children was 22 months, standard deviation (SD) 14 months. In the study group 23 months, SD 13 months and in the control group 21 months, SD 14 months.

**Figure 1:**
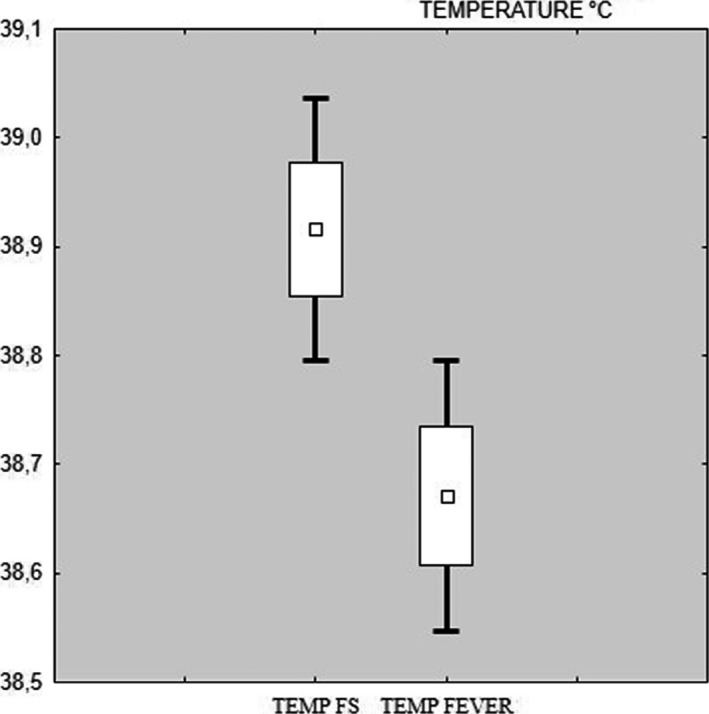
Box and whisker plot – temperature study (FS) and control (fever) group

In the study group mean body temperature was 39.0°C and in the control group 38.6°C. A statistically significant difference was found between body temperature of study and control group (*p = *.005) (Figure** 1).

**Figure 2:**
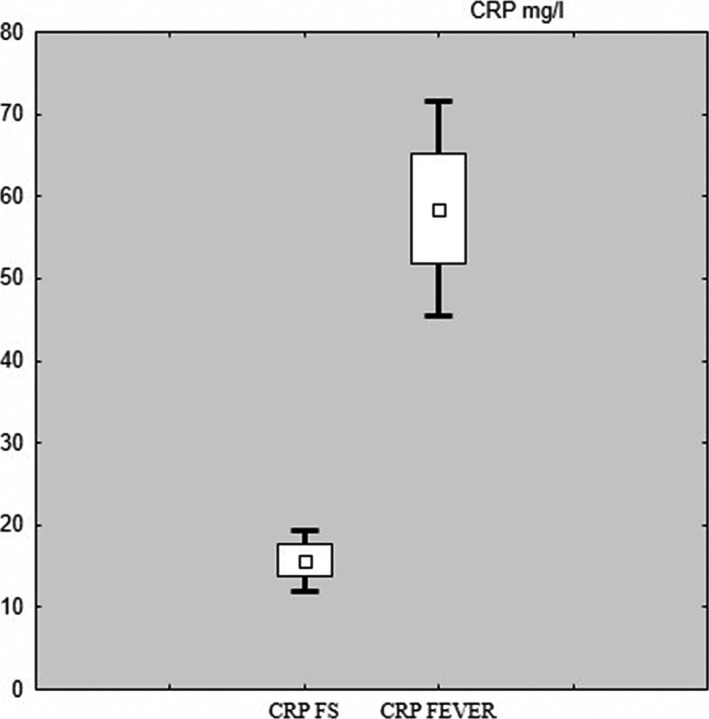
Box and whisker plot – CRP study (FS) and control (fever) group

The mean C‐reactive protein level in the study group was 15.73 mg/L and in the control group 58.20 mg/L. There was a statistically significant difference (*p < .001*) (Figure** 2).

**Figure 3:**
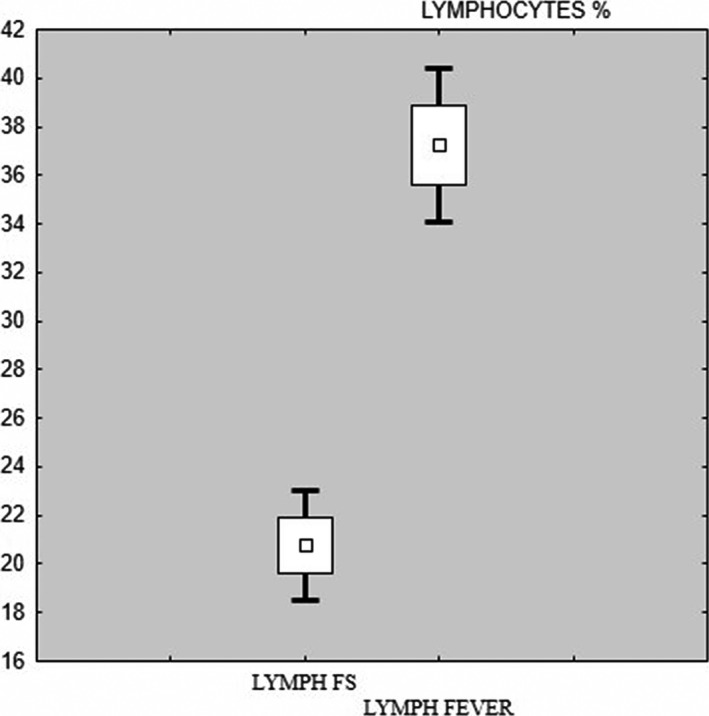
Box and whisker plot ‐ lymphocytes study (FS) and control (fever) group

**Figure 4:**
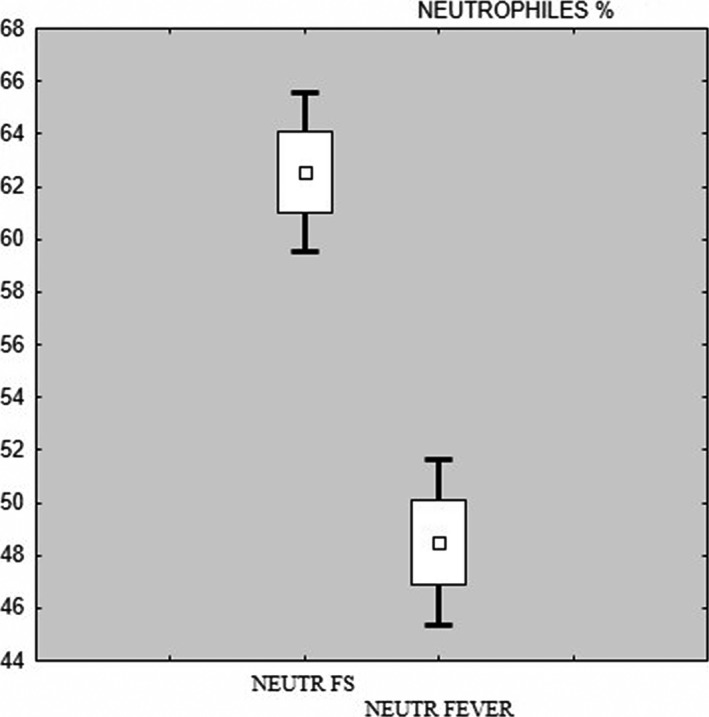
Box and whisker plot – neutrophils study (FS) and control (fever) group

In the study group mean neutrophil level was 62.55% and in the control group was 48.48%. In the study group mean number of lymphocytes was 20.77% and in the control group 37.22%. There was a statistically significant difference between the number of lymphocytes and neutrophils (*both p < *.001) (Figures** 3, 4). In statistical analysis, there was also a statistically significant difference between the number of hemoglobin, hematocrit and platelets (Table 1).

**Table 1:** Laboratory tests performed in children in the studied group and control group

	Study group		Control group		*p*
	Range of values	Mean	Range of values	Mean	
Temperature (°C)	37.0–40.6	39.0	37.1–40.3	38.6	**.005**
Blood cell count					
Leukocytes (G/L)	1.43–41.5	13.67	2.73–57.75	14.19	.564
Neutrophils (%)	8.4–89.9	62.55	5.5–85.5	48.48	**<.001**
Lymphocytes (%)	1.42–73.4	20.77	7.4–81.7	37.25	**<.001**
*Monocytes* (%)	1.38–38.7	12.91	3.7–22.54	12.89	.977
Hematocrit (%)	27.0–39.4	34.12	26.4–38.4	32.96	**<.001**
Hemoglobin (g/dl)	8.7–13.6	11.70	8.7–13.1	11.24	**<.001**
Blood platelets (thousands/ml)	124–561	303	134–963	341	**.005**
CRP (mg/L)	1.09–178.36	15.73	1.07–380.99	58.50	**<.001**

CRP, C‐reactive protein.

Bold text indicates statistical significance.

## 4 DISCUSSION

In this paper, authors analyzed laboratory results (BCC and CRP) in children presenting fever without seizures and FS who were admitted to hospital.

There were no differences in boys and girls number in the study group and the control group. Although there were more boys in the analyzed population of children. As in the papers of Yigit et al. (2017) and Goksugur, Kabakus, Bekdas, and Demircioglu (2014) occurrence of febrile seizures was higher in boys. Mean age of admitted children due to fever was 21 months, and in children with febrile seizures 23 months. Sharawat, Singh, Dawman, and Singh (2016) analyzing the same group of children with fever and febrile seizures mean age was 26.3 months vs. 24.9 months, with boys number significantly higher than girls (70% vs 30%).

Observation shows that in children with FS the body temperature was significantly higher than in children without FS (39.0 vs 38.6; *p = *.005). Higher body temperature (fever) in the FS group correlated with acute onset of symptoms of infection. Febrile seizures and high fever are characteristic for the high dynamic of infection development. Infection symptoms do not correlate with the increase of inflammatory factors. The peak of inflammatory markers such as CRP, procalcitonin, leukocytosis is slower than the clinical symptoms. Similar results were obtained in Sharawat et al. (2016), in which study body temperature was also significantly higher in children with FS compared to children with fever without seizures.

C‐reactive protein levels were significantly lower in children with FS compared to children without seizures (15.73 vs 58.50; *p < *.001). This can depend on the factor of infection that is responsible for significant body temperature rise in children, most commonly viruses. CRP reaches the highest values after 24–48 hr after the inflammatory response. Increasing is related to the presence of infection. It can be suspected that children with FS develop inflammatory processes and increase body temperature to very high values quickly enough that CRP levels do not reach their highest values. In children with fever without seizures, the inflammatory process was increasing slow enough to get CRP to higher levels (Bobilewicz, 2004; Całkosiński et al., 2009). Many papers evaluating BCC in children with fever and FS does not analyze inflammatory mediators. In the study, Yigit et al. (2017) mean CRP levels in children with simple and complex febrile seizures (14.92 mg/dl SFS vs. 19.3 mg/dl CFS) are comparable to the results obtained in this study. Goksugur et al. (2014) mean CRP in children with FS was higher than this paper result (25.47 mg/dl SFS vs 35.20 mg/dl CFS). They are still significantly lower than in the control group of the presented study.

In the presented study, neutrophil count was significantly lower in the group of children with fever without seizures than in the group of children with FS (48.48 vs. 62.55; *p < *.001). The number of neutrophils can temporarily and rapidly increase during intense skeletal muscle activity (e.g., seizures, chills), may be the result of an inflammatory reaction (after 4–5 hr) or may be associated with the presence of blood circulating toxins (Traczyk, 2002). In a study of Goksugur et al. (2014) mean neutrophil counts were slightly lower in children with FS compared to those reported in children with FS in this study. Researchers (Goksugur et al., 2014) did not show differences in the number of neutrophils between SFS and CFS group, which was demonstrated by Yigit et al. (2017). The authors observed that in children with SFS neutrophils were significantly lower compared to children with CFS (Yigit et al., 2017). Mean number of neutrophils in that studies were comparable to the results of this study (Goksugur et al., 2014; Yigit et al., 2017).

Children with FS had significantly lower lymphocytes levels in comparison to children with fever without seizures (20.77% vs 37.25%; *p* < .001). Higher levels of lymphocytes in the control group may be associated with more advanced inflammatory process. Children with fever without seizures were admitted to the hospital at the peak of the fever illness (with the highest body temperature value). It can be predicted that lymphocytes level was also higher than those in the FS group. For comparison, the mean number of lymphocytes in the study group in this study was significantly lower than the Yigit et al. (2017) and Goksugur et al. (2014). Authors (Goksugur et al., 2014; Yigit et al., 2017) have shown that lymphocytes were significantly higher in children with CFS compared to those with SFS.

The levels of hemoglobin and hematocrit were significantly higher in the FS group. Higher results may be due to dehydration of children with FS. Fluid deficit may cause cellular dehydration and electrolyte shift which decreased seizure threshold. Sharawat et al. (2016) did not find a correlation between hemoglobin value in the study group (with FS) and the control (with fever without seizures). On the other hand, in the results of studies presented by Yousefichaijan et al. (2014), authors found the same correlation between hemoglobin and hematocrit. Many authors point out that iron deficiency anemia in children is an important risk factor of the febrile seizures. This risk is higher in children with SFS between the ages of 6 months and 3 years (Derakhshanfar, Abaskhanian, Alimohammadi, & ModanlooKordi, 2012; Fallah, Tirandazi, Akhavan Karbasi, & Golestan, 2013; Kumari, Nair, Nair, Kailas, & Geetha, 2012; ur Rehman & Billoo, 2005; Sadeghzadeh, Khoshnevis Asl, & Mahboubi, 2012; Sherjil, us Saeed, Shehzad, & Amjad, 2010). A *Sherjil* et al. observed that the risk was doubled in children with FS compared to healthy children (Sherjil et al., 2010). No blood iron concentrations were analyzed in this study because test was not routinely ordered in admitted children.

The difference in the level of WBC in the FS group and the control group was not shown in the study. Yousefichaijan et al. (2014), results show that leukocytes were significantly higher in children with fever than with FS. These differences can come from group selection. Paper study group was consisted of children with SFS and first episode of seizures. At this study all children with FS no matter it was simple or complex or recurrent were investigated. In the above study, platelet count in children was significantly higher in the control group than in the study group, as confirmed by Yousefichaijan et al. (2014) Higher blood platelet counts may also be associated with blood clotting (there were also increase in hematocrit).

## CONFLICTS OF INTEREST

None.
